# Utilization potential of intraluminal optical coherence tomography for the Eustachian tube

**DOI:** 10.1038/s41598-021-85634-3

**Published:** 2021-03-18

**Authors:** Hayoung Byun, Yeon Hoon Kim, Jingchao Xing, Su-Jin Shin, Seung Hwan Lee, Hongki Yoo, Jae Ho Chung

**Affiliations:** 1 Department of Otolaryngology-Head and Neck Surgery, School of Medicine, Hanyang University, 222-Wangshimni-ro, Seongdong-gu, Seoul, 04763 Republic of Korea; 2 Department of Mechanical Engineering, Korea Advanced Institute of Science and Technology, 291 Daehak-ro, Yuseong-gu, Daejeon, 34141 Republic of Korea; 3 Department of Biomedical Engineering, Hanyang University, Seoul, Korea; 4 Department of Pathology, Gangnam Severance Hospital, Yonsei University College of Medicine, Seoul, Korea

**Keywords:** Imaging, Optical imaging, Medical imaging

## Abstract

Imaging the Eustachian tube is challenging because of its complex anatomy and limited accessibility. This study fabricated a fiber-based optical coherence tomography (OCT) catheter and investigated its potential for assessing the Eustachian tube anatomy. A customized OCT system and an imaging catheter, termed the Eustachian OCT, were developed for visualizing the Eustachian tube. Three male swine cadaver heads were used to study OCT image acquisition and for subsequent histologic correlation. The imaging catheter was introduced through the nasopharyngeal opening and reached toward the middle ear. The OCT images were acquired from the superior to the nasopharyngeal opening before and after Eustachian tube balloon dilatation. The histological anatomy of the Eustachian tube was compared with corresponding OCT images, The new, Eustachian OCT catheter was successfully inserted in the tubal lumen without damage. Cross-sectional images of the tube were successfully obtained, and the margins of the anatomical structures including cartilage, mucosa lining, and fat could be successfully delineated. After balloon dilatation, the expansion of the cross-sectional area could be identified from the OCT images. Using the OCT technique to assess the Eustachian tube anatomy was shown to be feasible, and the fabricated OCT image catheter was determined to be suitable for Eustachian tube assessment.

## Introduction

The Eustachian tube is an extended part of the upper respiratory tract, which connects the middle ear space and the nasopharynx^1^. As the only route for ventilating air spaces in the middle ear and mastoid cavity, the complex valve function of the Eustachian tube plays an important role in maintaining middle ear homeostasis, that is, air pressure equalization between the middle ear cavity and atmosphere, middle ear oxygenation, draining of secretions from the middle ear, and protection of the middle ear from retrograde infections^2,3^. The Eustachian tube consists of complex structures of intratemporal bone and a fibrous, cartilaginous portion. The proximal one-third—the osseous portion—is a funnel-shaped bony extension of the middle ear cleft. The distal two-thirds—the pharyngeal part—consists of a cartilaginous skeleton attached to several tubal muscles capable of dynamic movements^1^. By autonomically controlling the peritubal muscles, the Eustachian tube opens and closes to ventilate and protect the middle ear cavity. Despite the clinical importance of dynamic Eustachian tube evaluation, physical examination of the Eustachian tube is challenging due to its location in the parapharyngeal space of the infratemporal fossa^4^.

Dysfunction of the Eustachian tube has been suggested as an etiology of various middle ear pathologies, although the underlying mechanism has remained poorly understood^5^. The dynamic function of the tubal structures has been an issue of interest in the otology fields, but their limited anatomical accessibility has been an obstacle to proper evaluation. As the only non-invasive route to access the Eustachian tube, the nasopharyngeal opening has been used for direct inspection of the Eustachian tube. Various imaging modalities and techniques have been employed to assess the Eustacian tube’s morphology and function, including endoscopy, radiography, computed tomography, ultrasound, and so on^6–8^. However, feasible imaging techniques capable of reflecting the physiologic function of the Eustachian tube are still lacking^2^.

Optical coherence tomography (OCT) is a novel, non-invasive imaging technique that can provide real-time images with a microscopic resolution of 10–20 μm. It has been widely used in the ophthalmology field^9,10^, and since the development of a luminal OCT system, OCT application has been expanded to the diagnosis of coronary artery problems and esophageal disease^11–16^. As a specific example, Schuon et al. conducted an ex vivo experiment to acquire a Eustachian tube image with a coronary artery OCT system^17^. However, previously developed luminal OCT systems are not suitable for Eustachian tube assessment. For example, an OCT system designed for the esophagus is too large for the Eustachian tube, and a coronary OCT system not only requires a guidewire for OCT placement, it also does not provide images near the tip of the OCT catheter, which could make procedures in the Eustachian tube difficult.

In the present study, we aimed to fabricate a unique OCT imaging catheter suitable for Eustachian tube evaluation, and to evaluate the feasibility for assessing the Eustachian tube anatomy by serial histology matching with swine cadavers.

## Materials and methods

### Development of an OCT system suitable for Eustachian tube examination

An OCT catheter consists of a transparent catheter sheath and an imaging probe with a side-viewing ball lens that emits near-infrared light. The OCT measures the echo time delay and signal intensity after its reflection or back-scattering from the tubal wall structures while the imaging probe rotates and moves backward (pullback) inside the catheter sheath to obtain real-time cross-sectional images.

**Figure 1:**
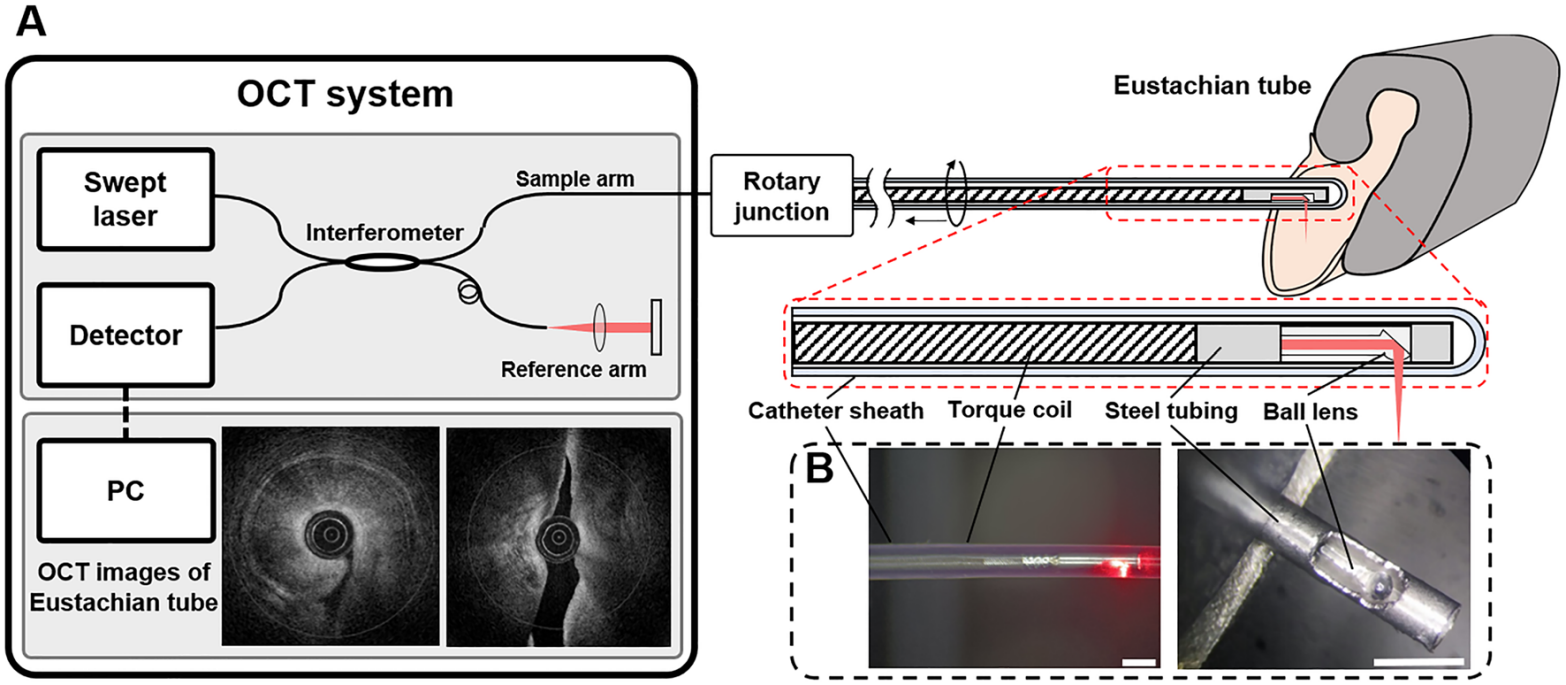
Experimental setup of the Eustachian optical coherence tomography (OCT) system (**A**). Photographs of the OCT catheter (**B**), showing the side view of the catheter (**B**, left) and the distal end of the imaging probe without the catheter sheath (**B**, right).

We designed a unique optical image catheter suitable for studying the Eustachian tube, referred to as a Eustachian OCT, by modifying the catheter system used for coronary arteries (Fig. 1A). The specialized round-tipped OCT catheter was fabricated with a catheter sheath with 1.01 mm outer diameter, 0.71 mm inner diameter, and 2.0 mm distance between the tip of the catheter sheath and the imaging probe, to minimize the blind area (Fig. 1B). To enhance the resolution and OCT penetration depth, the imaging catheter was made of fluorinated ethylene propylene (FEP), which has a lower refractive index (1.33) that is more similar to water than Pebax(1.508), a commonly used material in coronary artery imaging. For imaging the Eustachian tube, we used a prototype swept-source OCT system (NinePoint Medical, Cambridge, MA, USA) with a central wavelength of 1300 nm and a bandwidth of 100 nm, resulting in a measured axial and lateral resolution of 8.27 $\mathrm{\mu m}$ and 22.67 $\mathrm{\mu m}$, respectively. The imaging part consisted of a custom-built rotary joint (Princetel, Inc, NJ, USA) and the imaging catheter. Using a rotation motor (Faulhaber, Croglio, Switzerland) and a translational stage (Zaber, Vancouver, Canada) connected within the rotary joint, the imaging probe with 0.6 mm outer diameter rotated inside the catheter sheath and moved backward to acquire three-dimensional OCT images of the luminal structures. The settings for the image acquisition were a pullback speed of 3 mm/s, a frame rate of 50 frames/s, and a pullback length from 35 to 45 mm. The acquired images were reconstructed to show cross-sectional and longitudinal images of the Eustachian tube.

### Animal handling protocol

The present study used three fresh cadaver heads of male swine obtained from a slaughterhouse (Daejeon, Korea). The swine heads were divided according to the sagittal plane, and then the posterior nasal septum was removed to visualize the pharyngeal opening of the Eustachian tube. Further resection was performed leaving the structure around the Eustachian tube, external auditory canal, mastoid bone, and tympanic bone for image acquisition and histologic slide preparation. The Eustachian tube lumen was irrigated with saline through the nasopharyngeal opening using a 50 cc syringe with a 24-gauge plastic needle to remove mucous materials in the lumen.

### Acquisition of ex vivo images of the Eustachian tube

**Figure 2:**
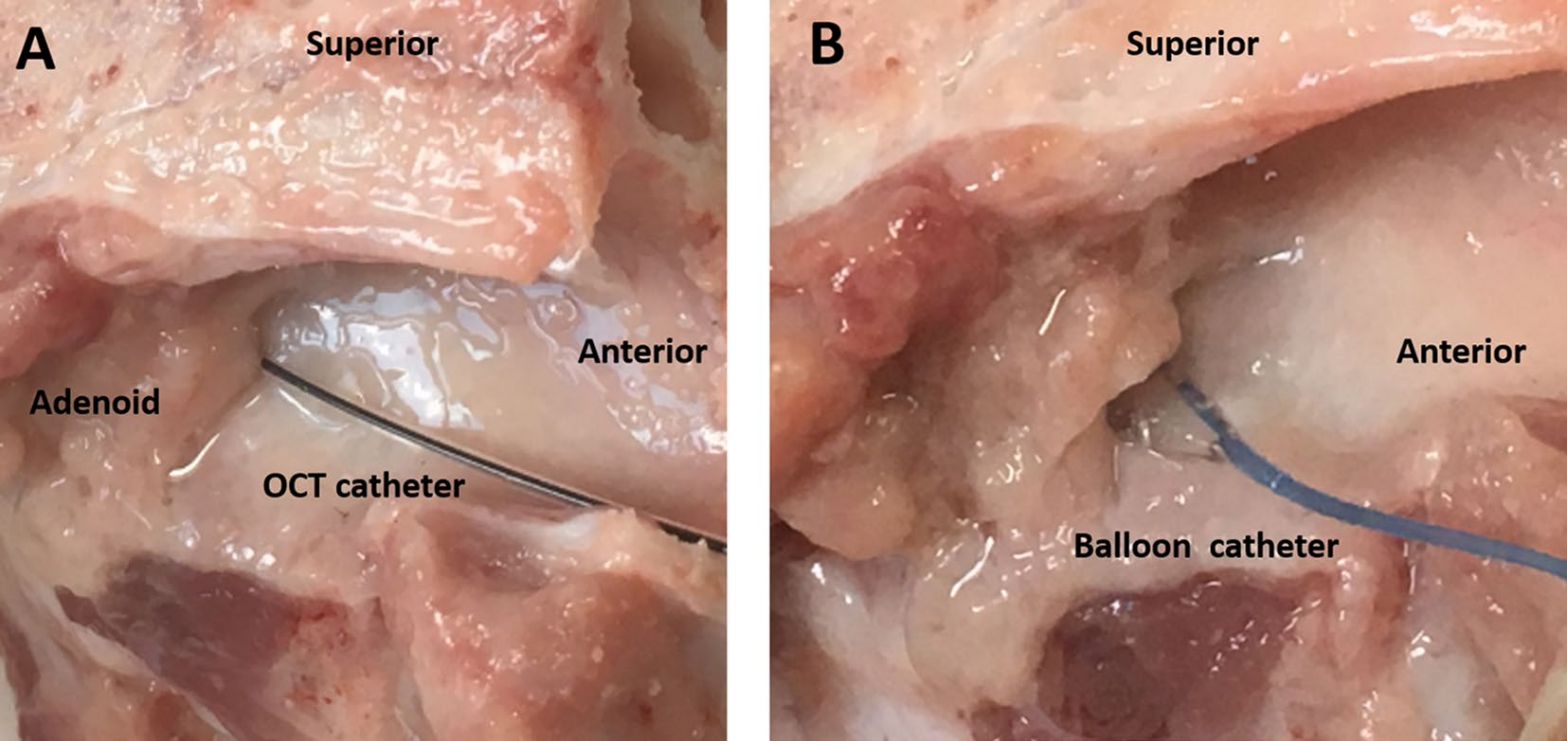
Placement of the optical coherence tomography catheter (**A**) and balloon catheter (**B**) through the left nasopharyngeal opening of the swine Eustachian tube.

The OCT catheter was introduced into the swine Eustachian tube through the nasopharyngeal opening. The catheter entered the pharyngeal portion of the Eustachian tube without resistance. When the catheter tip had reached about 35 mm inside, resistance was felt in the isthmus portion of the tube. If the catheter passed through the isthmus, the catheter was inserted at about 0.5 mm further to avoid damage to the middle ear structures. After confirming the OCT catheter was placed in the proper position, image acquisition was performed by helical pullback scanning of the imaging probe (Fig. 2A).

After taking resting state images of the Eustachian tube, an Eustachian tube ballooning procedure was performed. An angioplasty balloon catheter was introduced in the nasopharyngeal opening of the Eustachian tube under direct visual observation. The balloon was inflated to a pressure of 2 bar for 10 min (Fig. 2B). After the balloon dilatation procedure, the OCT catheter was reinserted through the nasopharyngeal opening, and the images were acquired.

### OCT image processing and interpretation

We obtained 500–700 frames of OCT cross-sectional images from a 35–42-mm-long Eustachian tube. Then, the three-dimensional (3D) images were reconstructed by combining all the OCT cross-sectional images with a frame interval of 60 µm. Longitudinal sections were also generated by cutting the 3D images laterally. Image processing software (ImageJ 1.52, National Institute of Health, Bethesda, MD, USA) was used to manage the cross-sectional and longitudinal images. Volume rendering software (Osirix, Pixmeo SARL, Geneva, Switzerland) was used to generate the 3D images and the cut-away longitudinal images. An experienced OCT scientist and an otolaryngologist interpreted the 3D OCT images based on the histologic anatomy of the Eustachian tube.

To compare the luminal area before and after balloon dilatation, we manually segmented the luminal area and counted the number of pixels in order to convert them into a physical area. The luminal areas obtained from the cross-sectional frames were compared at each longitudinal position.

### Preparation of histological cross-sections of the Eustachian tube

After acquisition of the OCT images, a 24 gauge angio-catheter was inserted through the nasopharyngeal opening to track the lumen of the Eustachian tube during the histological preparation procedures. The specimen was washed in saline solution and fixed in formalin. After the decalcification process, serial cross-section slides were prepared by cutting specimens in a plane perpendicular to the previously inserted catheter, from the nasopharyngeal opening to the middle ear cavity at approximately 7 mm intervals. All specimens were stained with hematoxylin and eosin (H&E) and the Eustachian tube lumen was traced. Finally, the cross-sectional images of the OCT were compared with histological cross-sections at the corresponding position.

### Ethics approval

This study was approved by the Institutional Animal Care and Use Committee of Hanyang University 2019-0040A).

## Results

### OCT images of the Eustachian tube

The Eustachian OCT catheter was successfully introduced into the Eustachian tube without trauma through the nasopharyngeal opening. In one of the three swine heads, the OCT catheter passed the isthmus with only slight resistance, and the tip entered the middle ear space without difficulty. In the other two swine heads, the OCT catheter stopped at the isthmus portion and was unable to advance, though there was no catheter breakage or bending during insertion.

**Figure 3:**
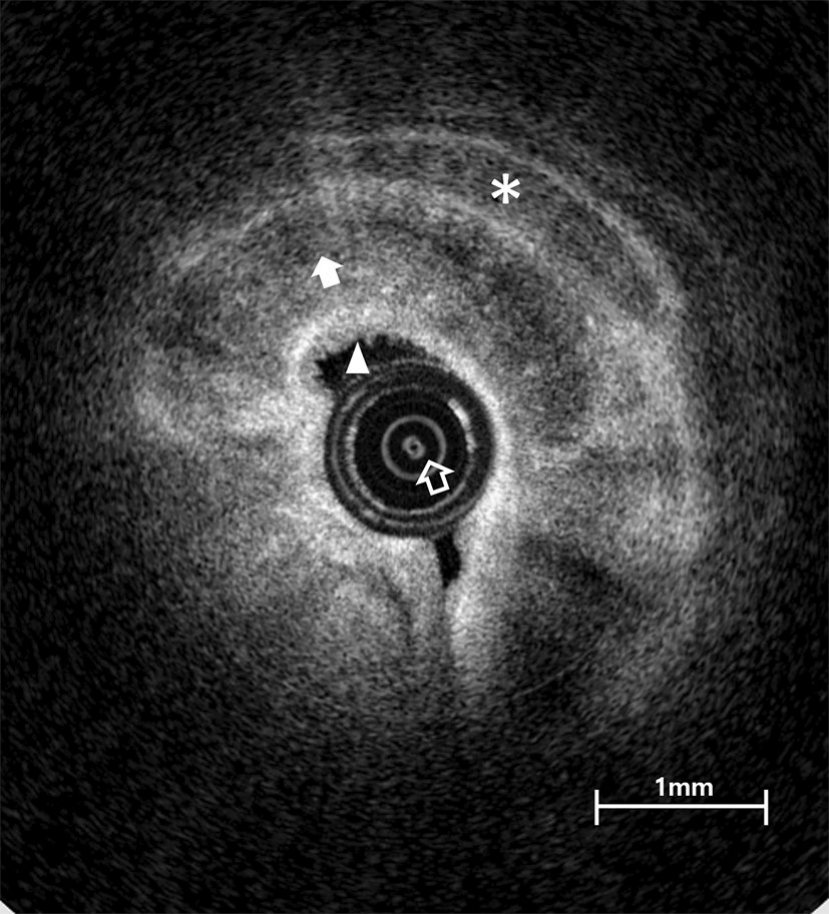
A representative optical coherence tomography image from near the isthmus portion of the left Eustachian tube, identifying cartilage (white filled arrow), mucosa (filled arrowhead), inner imaging catheter (empty arrow), and fat layer (asterisk).

A representative OCT image of the Eustachian tube and adjacent structures are shown in Fig. 3. The margin of the shepherd’s crook-shaped cartilage, adjacent fat tissues, and luminal space could be delineated at the proximal portion of the Eustachian tube near the isthmus. The mucosal surface of the Eustachian tube and submucosal tissue were also distinguished.

**Figure 4:**
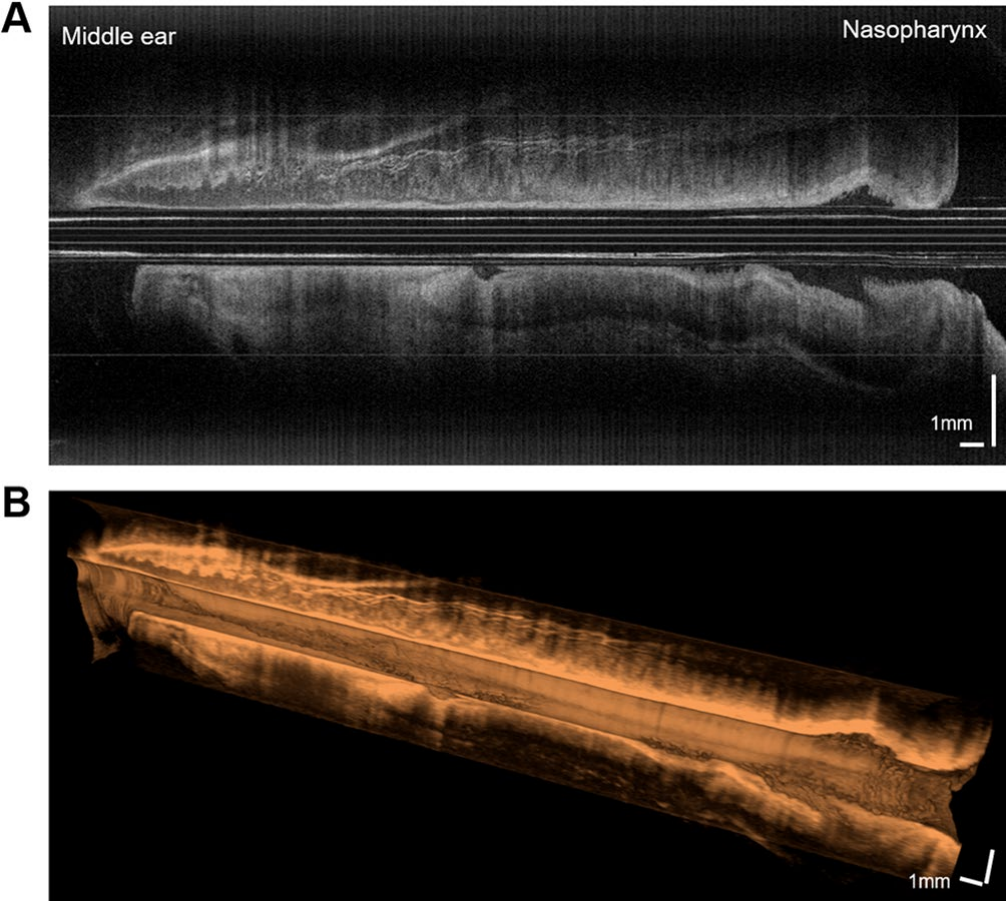
Longitudinal images of the Eustachian tube. The mucosal layer and peritubal structures are shown for the entire length of the tube (**A**). A 3D cut-away longitudinal image reveals the surface lining of the Eustachian tube (**B**).

The reconstructed longitudinal image of the whole Eustachian tube showed the continuous lining of the respiratory epithelium and underlying structures, as seen in Fig. 4A. The cut-away image of the Eustachian tube successfully delineates its epithelial surface (Fig. 4B).

### Histologic correlation of the OCT image

**Figure 5:**
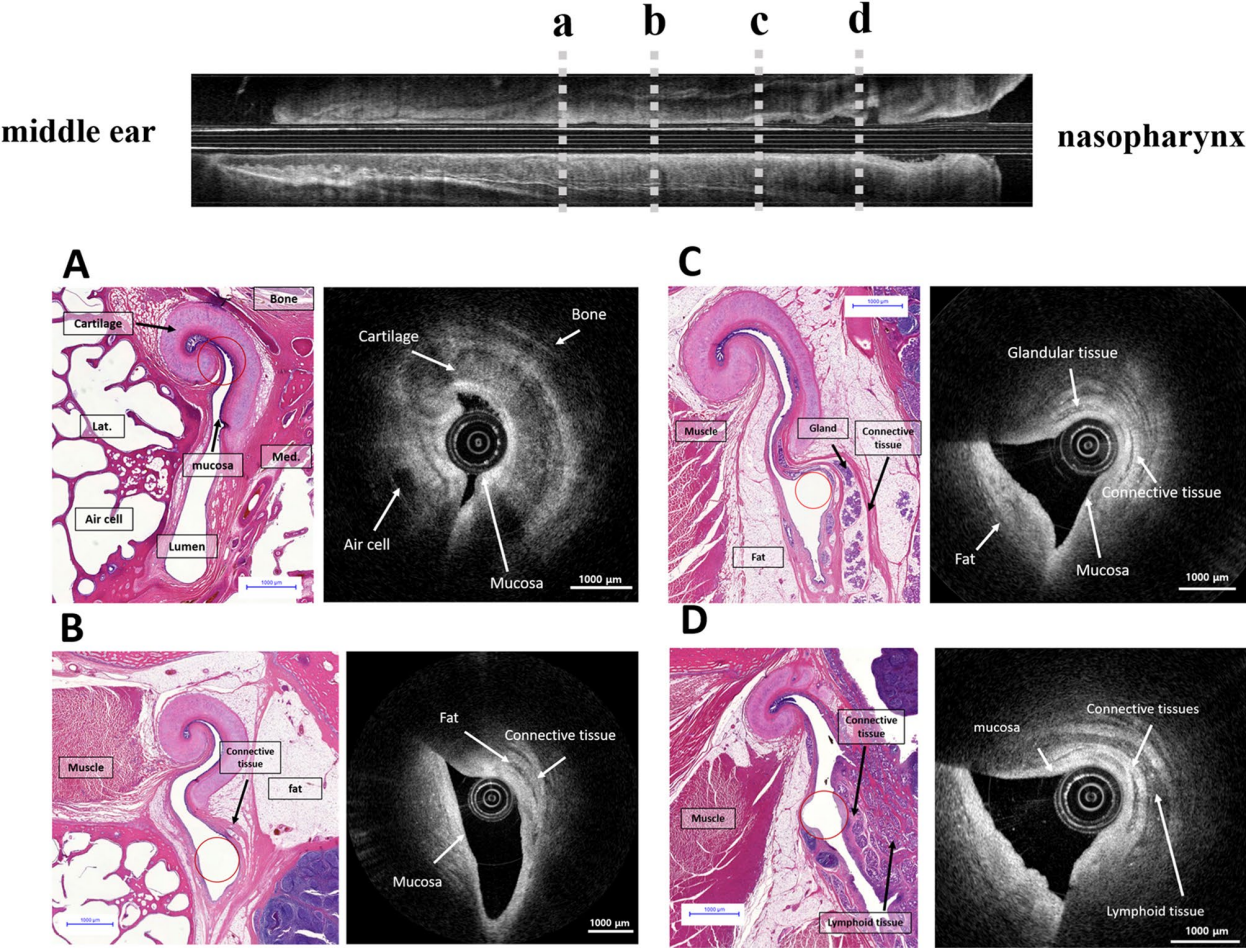
Histologic images matched with the optical coherence tomography (OCT) images of the left Eustachian tube from the isthmus portion (**A**) to the nasopharyngeal opening (**D**). The hook-shaped Eustachian tube cartilage can be observed at the cartilagenous portion of the Eustachian tube near the isthmus, and the mucosa of the Eustachian tube was identified (**A**). Low reflective structures of the peritubular muscle and fat tissue were identified in both the OCT images and the corresponding histological section (**B**). The tubal mucosa, submucosal glandular structures, adipose tissue, and connective tissue were distinguished in the OCT image (**C**). Nasopharyngeal lymphoid tissues were seen in the nasopharyngeal inlet of the Eustachian tube (**D**). The position of the OCT catheters are indicated by empty red circles in the histologic images.

A comparison of the serial images in the histologic slides and OCT scans are presented in Fig. 5. The morphological difference in the tubal cartilage is noticeable as it goes down from the bony-cartilage transition portion. The hook-shaped Eustachian tube cartilage can be identified, and the mucosal lining of high reflection (bright in the OCT image) can also be observed near the cartilage portion of the Eustachian tube near the isthmus (Fig. 5A). High-reflective connective tissues and a low-reflective (dark in the OCT image) fat layer could be identified in both the OCT images and the corresponding histological section (Fig. 5B). In the inferior part of the Eustachian tube near the nasopharyngeal opening (Fig. 5C, D) submucosal glandular structures, a fat layer and lymphoid tissue are abundantly observed. The tubal mucosa, submucosal glandular structures, connective tissues, and adipose tissue are distinguished in the OCT image of the inferior portion (Fig. 5C). Nasopharyngeal lymphoid tissues can be identified in the OCT image as multiple spots of high reflection in the low-reflection background at the nasopharyngeal inlet of the Eustachian tube (Fig. 5D).

### Changes in the luminal area after Eustachian tube ballooning

**Figure 6:**
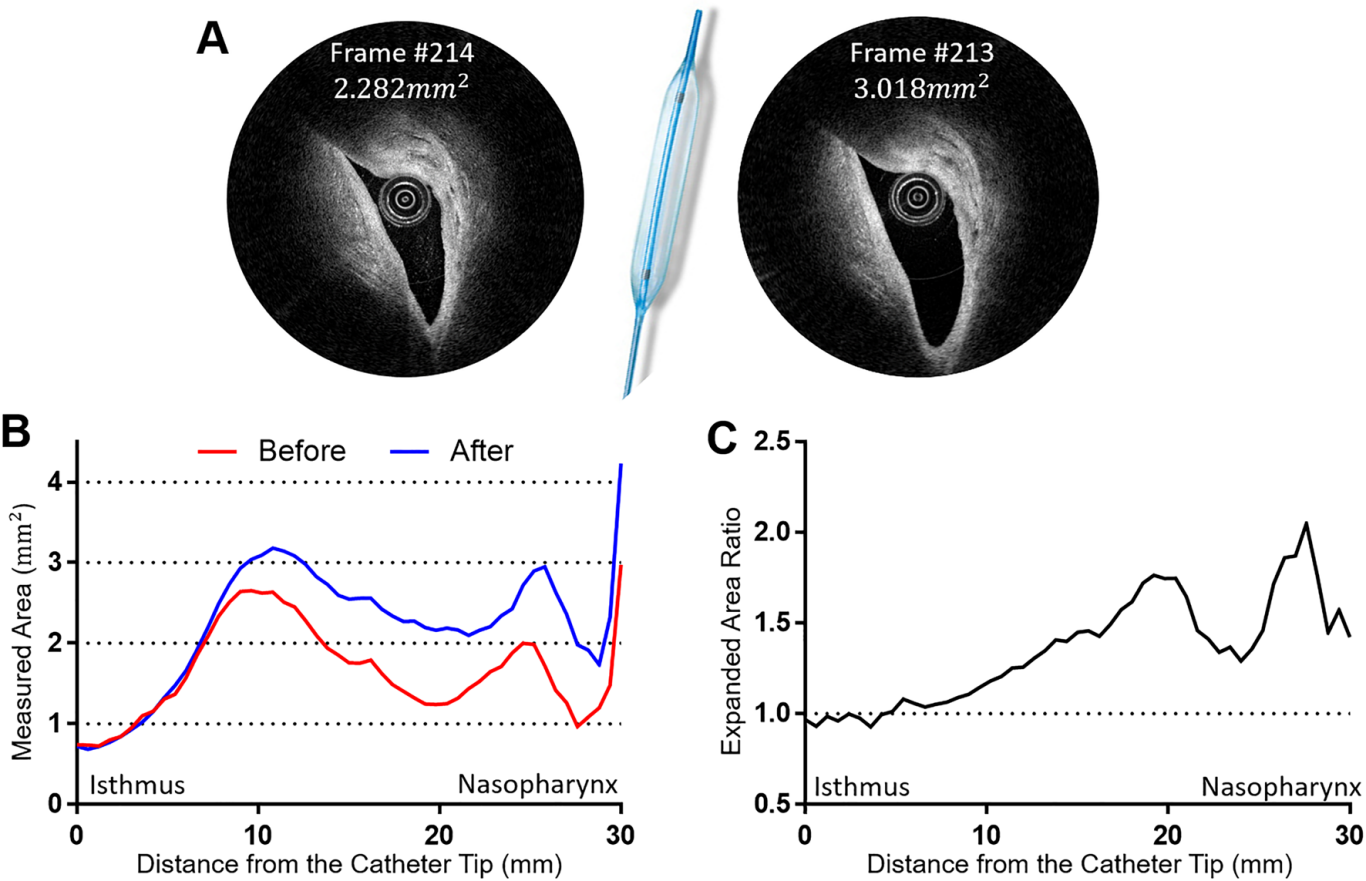
Changes in the luminal area before and after balloon dilatation of the Eustachian tube. The cross-sectional area was calculated by converting the pixels (per inch) into areas (mm^2^). The areas of the same location were compared before and after balloon dilatation. In the mid-portion of the Eustachian tube (**B**), the luminal area increased from 2.282 to 3.018 mm^2^ (**A**). The widest luminal area was measured at around 10 mm inferior from the isthmus (**B**). The luminal area ratio before and after balloon dilatation tended to increase as it moved toward the nasopharyngeal opening of the Eustachian tube (**C**). The a, b, c, and d in (**B**) indicate corresponding locations presented in Fig. 5.

Images of the luminal area before and after Eustachian tube ballooning were compared (Fig. 6). The cross-sectional area was calculated by converting the pixels (per inch) into areas (mm^2^). Figure 6A shows the change of luminal area at the same portion of the Eustachian tube before and after balloon dilatation. The increase in the Eustachian tube luminal area was more evident near the nasopharyngeal orifice than near the isthmus portion (Fig. 6B), with the maximum ratio of area change being 2.05 in the nasopharyngeal portion (Fig. 6C). The mean expanded area ratio was 1.35 ± 0.29, and the expanded area ratio tended to increase as it moves toward the nasopharyngeal opening of the Eustachian tube (Fig. 6C).

## Discussion

In the present study, we assessed the feasibility of Eustachian tube imaging using a newly fabricated Eustachian OCT catheter. The results can be summarized as follows: (1) The OCT catheter was successfully introduced through the nasopharyngeal opening of the Eustachian tube without mucosal damage, (2) real-time OCT images could be acquired and the major structures of the Eustachian tube could be identified in the OCT images, and (3) the effect of the Eustachian tube balloon dilatation could be objectively measured with the OCT images.

It is recognized that OCT is a type of imaging technique that measures the scattering and reflection of near-infrared light to provide real-time high-resolution cross-sectional tomographic images of microstructures^9^. The advantage of OCT images is their high spatial resolution, of 10–20 μm, and quick acquisition time. However, the depth of an OCT image is limited to around 1–3 mm below a surface due to optical attenuation by tissue scattering and absorption. The technique of OCT has been applied in various clinical settings to diagnose diseases or monitor pathological processes^18^. In ophthalmology, an OCT test is included in the routine ophthalmological examination battery to assess the retinal anatomy and related pathological processes^19^. In addition, a benchtop OCT system and a customized probe can be used in dermatology and dentistry to evaluate the microstructures in skin epithelium, enamel, dentin, or gingival mucosa^20,21^. In addition, a catheter-based luminal OCT technique has been used to evaluate tubular structures, including coronary arteries and the urinary and digestive tracts^11–16,22,23^. The coronary OCT system can capture the status of arteriosclerotic plaque and pathologic changes in coronary artery walls, and subepithelial structures of the esophagus and gastrointestinal wall can be evaluated with OCT-incorporated endoscopy^15^.

As an illustration of how the application of OCT has expanded, one study attempted to image the Eustachian tube in a sheep cadaver head with a commercially available coronary OCT catheter (Dragonfly Duo catheter)^17^. The researchers of that study successfully inserted the OCT catheter into the Eustachian tube and acquired a representative image of tubal cartilage. Although the results of that study support the viability of Eustachian OCT technology, it was acknowledged that it was necessary to modify the coronary OCT catheter because the catheter tip had a long non-functioning radiopaque area 20 mm in length. In order to observe the entire Eustachian tube, the coronary catheter needed to be inserted 20 mm further into the middle ear, which risked damage to the ossicular chain in the middle ear.

To overcome such issues, the current study adopted the high-resolution OCT imaging technique and fabricated a specialized catheter system for use in the Eustachian tube. It was important to minimize the distance between the tip of the catheter sheath and the imaging probe, the blind area, to obtain images of the entire Eustachian tube without middle ear damage. The result was a new OCT catheter with a 2.0 mm long blind area with a rounded tip (Fig. 1). Determining the appropriate diameter of the catheter sheath was another critical issue, since it is important to enable proper insertion without mucosal trauma or catheter bending. In this study, the outer diameter of the catheter was gradually reduced from 1.20 to 0.74 mm to ease catheter advancement through the narrow Eustachian tube. The thinner catheters, however, had problems with breaking or collapsing during the insertion procedure, so the diameter of 1.01 mm was chosen as the most effective thickness. We also replaced the material of the catheter sheath to enhance image acquisition depth from the OCT catheter. The present study used fluorinated ethylene propylene, whose refractive index is more similar to water than the refractive index of those commonly used in the coronary artery OCT imaging catheter.

The microstructure of the Eustachian tube could be successfully identified in the real-time OCT images, due to differences in relative reflections depending on the tissue characteristics around the Eustachian tube (Figs. 3 and 5). As demonstrated in earlier work, fat, skin, and cartilage show different tissue characteristics in OCT images^24^. Tissue cellularity and water composition are important factors in determining the penetration, absorption, and reflection of light, leading to tissue discrimination. These tissue features provide the boundaries of structures in OCT images^17,24^. Respiratory epithelium with high cellularity could be characterized by high-intensity reflection, which appeared as bright areas in the OCT images^17,25–27^. Similarly, in our results, high cellularity regions of mucosal epithelium showed higher reflection on the OCT scan, while low-cellularity regions of fat, muscle, cartilage, and bone showed lower reflection^24^. In addition, glandular cells and lymphoid tissue were identified as multiple high-reflective spots in the background of low reflection (Figs. 3 and 5), and dense connective tissues showed higher reflection, revealing the boundaries of those structures (Fig. 5C,D).

In OCT images of the superior part of the Eustachian tube, the mucosa and cartilage structure were mainly outlined (Fig. 5A), while the peritubal muscle and fat pad covered with mucosa were identified in the lower portion of the tube (Fig. 5D). Due to an hourglass-like luminal space of the Eustachian tube, the lumen could be entirely observed near the narrow isthmus portion, while the wider inferior area could only be partially evaluated with the OCT (Fig. 5). That is, the position of the imaging catheter in the lumen can affect the acquired OCT images, and therefore a comprehensive understanding of the peri-tubal structure is needed to properly interpret the images.

The current study objectively showed the effect of balloon dilatation of the Eustachian tube, which is an emerging treatment modality for Eustachian tube dysfunction. After balloon dilatation, the OCT catheter was reinserted into the two swine Eustachian tubes, where the catheter had not previously passed through the isthmus portion, and it was observed to advance into the middle ear cavity without difficulty after the ballooning procedure. In addition, the cross-sectional area of the lumen was increased by various degrees after balloon dilatation (Fig. 6). The luminal area near the isthmus did not change after ballooning, while the cross-sectional area near the nasopharyngeal opening expanded by up to double. The isthmus portion of the Eustachian tube could not be dilated, not only because of the bony surrounding structures but also due to the limited placement of the balloon catheter through the isthmus. The various effects of balloon dilatation in portions of the Eustachian tube other than the isthmus may result from the changes in cartilage shape along the longitudinal axis of the Eustachian tube. Accordingly, it is expected that the Eustachian OCT system could be a useful tool for assessing the outcome of balloon dilatation and could be used for designing a customized balloon that is optimized for the Eustachian tube.

The present study successfully fabricated a unique Eustachian OCT catheter and interpreted OCT images by matching them with serial histologic sections. With OCT image analysis, the luminal changes of the Eustachian tube after the ballooning procedure in an ex vivo setting could be objectively measured. However, among the limitations of our results, the Eustachian tube could not be assessed in a living animal due to the narrow and complex anatomy from the nostril to the Eustachian tube opening. Although the size of the imaging catheter was small enough to pass through the tubal lumen and isthmus, there was concern about the Eustachian tube OCT images in this study in that the dilated state could be passively caused by the catheter itself. Consequently, several technical issues have to be solved to be able to apply this technique in a clinical setting. A guiding instrument to introduce the OCT catheter into a nasopharyngeal orifice is required to facilitate transnasal insertion for a human Eustachian tube. In addition, technical consideration is needed so as not to deform the catheter sheath under bending or the pressure ouside the sheath, which interfere with the rotation or pullback of the imaging probe inside the sheath during image acqusition.

We expect our Eustachian OCT system can be further modified and applied in clinical practice in the future. The OCT could observe and image not only static tubal structures but also real-time dynamic movements during swallowing and the Valsalva maneuver. When the balloon-incorporated Eustachian OCT catheter is fully developed, the Eustachian tube can be monitored in real time during balloon dilatation procedures.

## Conclusions

The present study fabricated a novel luminal OCT image catheter setup suitable for Eustachian tube assessment. An ex vivo experiment was conducted to demonstrate the use of the OCT in swine Eustachian tubes. The Eustachian OCT catheter was successfully introduced through the nasopharyngeal opening and progressed to the middle ear cavity, and OCT images of the Eustachian tube were acquired. Serial correlation of the histology slides and corresponding Eustachian tube OCT images confirmed the validity of the Eustachian tube structures represented as OCT images. In addition, luminal expansion of the Eustachian tube was objectively identified with OCT images after balloon dilatation. We expect the fabrication of the Eustachian OCT catheter to be an initial step in the clinical application of OCT in Eustachian tube assessment.
